# A Smartphone App for Improving Clinical Photography in Emergency Departments: Comparative Study

**DOI:** 10.2196/14531

**Published:** 2019-07-31

**Authors:** Chung-Hsien Liu, I-Chun Lin, Jui-Jen Lu, Dengchuan Cai

**Affiliations:** 1 Graduate School of Design National Yunlin University of Science and Technology Yunlin Taiwan; 2 Department of Emergency Medicine Ditmanson Medical Foundation Chiayi Christian Hospital Chiayi Taiwan; 3 Department of Industrial Engineering and Management National Yunlin University of Science and Technology Yunlin Taiwan

**Keywords:** smartphone app, health informatics, clinical photography, patient privacy

## Abstract

**Background:**

Digital photography is crucial for electronic medical records (EMRs), particularly for documenting dermatological diseases and traumatic wounds. In modern emergency departments (EDs), digital cameras are commonly used for photography, but the process is time-consuming. The problems of addressing patient privacy issues and that of interruptions and heavy workloads can cause archival errors when uploading photos. However, smartphones are widely available and cheap, so with a suitable app many errors could be mitigated.

**Objective:**

The aim of this study is to design and test a smartphone app to improve the efficiency of clinical photography and improve patient privacy in the ED. The app is connected to the hospital information system to verify patient identification and enable archiving, and the app can automatically delete images after upload to the patient’s EMR.

**Methods:**

This study enrolled 48 experienced ED nurses trained in clinical photography. Each nurse was first assigned a digital camera for photography and then a smartphone with the app preinstalled after it was launched. The time taken to upload images to a patient’s EMR was then recorded and the efficiency of the digital camera and app groups were compared.

**Results:**

The average time taken to upload images to a patient’s EMR for the camera and app groups were 96.3 s (SD 19.3; *P*<.001) and 26.3 s (SD 4.7; *P*<.001), respectively.

**Conclusions:**

The app effectively reduced processing time and improved clinical photography efficiency in the ED. Some issues of patient privacy in the camera group were revealed and resolved in the app group.

## Introduction

A picture is said to be worth a thousand words, which may now also be true in medical diagnoses. Patient photography has changed the way health care providers (HCPs) document, discuss, and deliver modern medical care. Medical photography is frequently used for the following: (1) documentation and consultation; (2) education; (3) patient and family instruction; and (4) journal publications [1]. In emergency departments (EDs), traumatic injuries and dermatological diseases are frequently recorded photographically first [2]. The Taiwanese National Health Insurance (NHI) administration regulates photographic records created by hospital staff to assess corresponding payments and treatment. Photography is also used to record and document healthcare issues for legal and judicial applications [1,2]. Because of the rapid development of information technology (IT) systems in healthcare, photographic film is not used in modern hospitals. Electronic medical records (EMRs) provide a suitable tool for communication within care teams and tracking of patients. Because clinical photography only has to record the actual condition of patients, even nonprofessional photographers can create records using photography in EDs [3].

Although digital images are becoming increasingly popular, several concerns regarding patient privacy need to be addressed [4,5]. From the perspective of patients, hospital-owned photographic devices are preferred over personal devices [5]. Digital cameras are currently the most common method of recording and uploading images to EMRs in hospitals. EMR systems typically have strict IT security to approve, restrict, and record the access of users, so the connection of cameras and transmission of images have consequently become somewhat time-consuming processes. Occasionally, archival errors can occur, and patient safety and privacy can be endangered. In addition, clinicians need to delete photos on the capture devices manually to prevent a privacy breach, which also takes time [6].

Modern smartphones with high quality cameras and software are now highly advanced and are more similar to laptops or handheld computers. A market for smartphone applications devoted to healthcare is emerging [7,8], and many apps serve numerous users in various fields, including clinical practice, medical education and patient instruction [9]. Medical devices and apps are already invaluable tools for HCPs, and as the range of features and uses expands, they are expected to exhibit greater market penetration in all aspects of clinical practice [10].

Smartphones are already competing strongly against single-purpose cameras in the photography market. However, unlike most digital cameras, the transmission capabilities of smartphones make them highly vulnerable to privacy breaches [11,12]. Leveraging the benefits of smartphones while avoiding their vulnerability to privacy breaches would be a considerable development in healthcare. In this study, we reviewed the research and designed an app that can reduce the time required to record and upload images as well as reduce archival errors. Patient confidentiality and privacy improvements are also discussed.

## Methods

### Application Design

**Figure 1:**
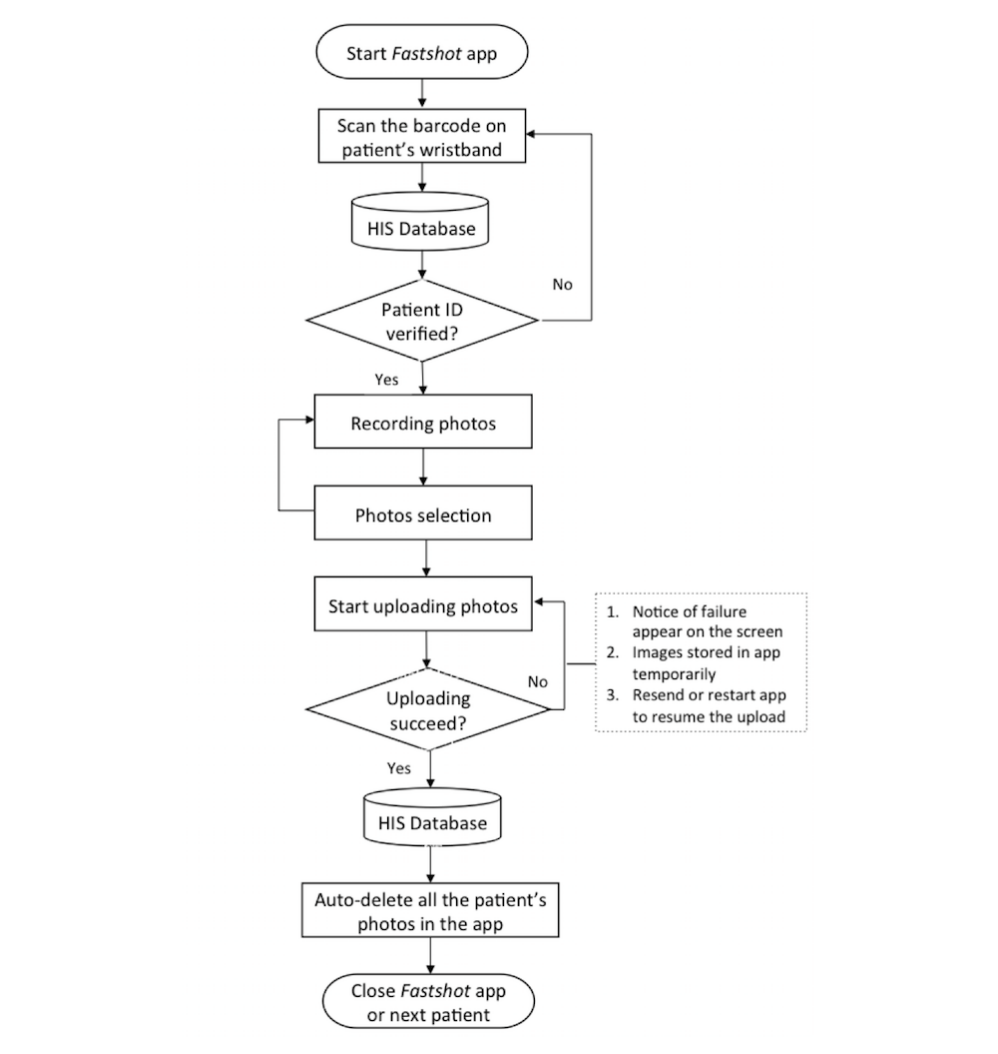
The workflow of the functional steps of the Fastshot app. HIS: hospital information system; ID: identification.

**Figure 2:**
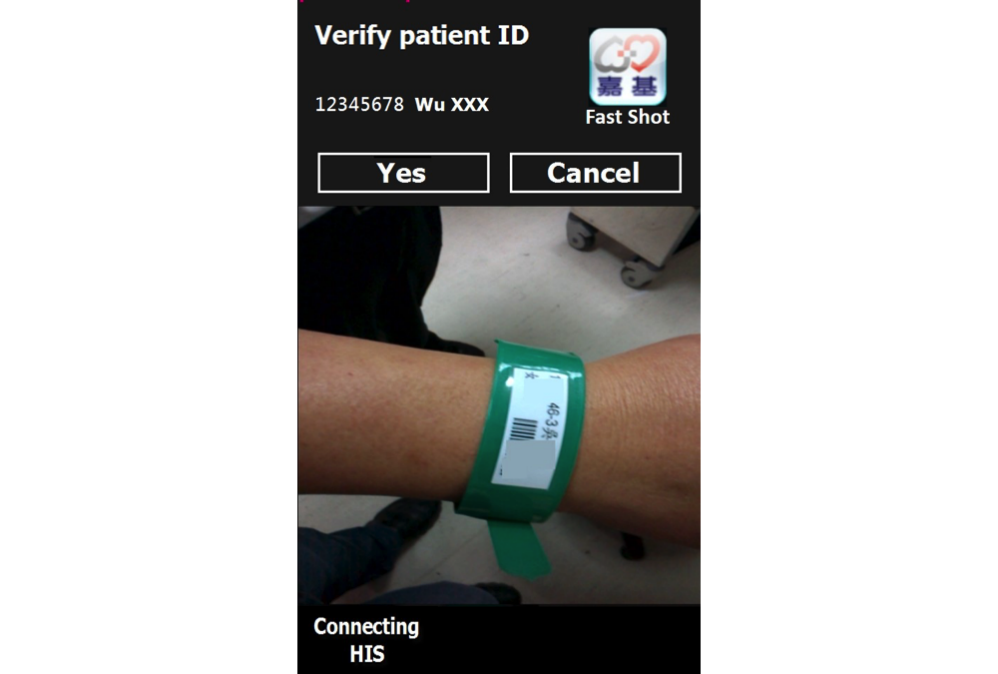
Scan a wrist band and connect to hospital information system to verify patient identity. HIS: hospital information system. ID: identification.

**Figure 3:**
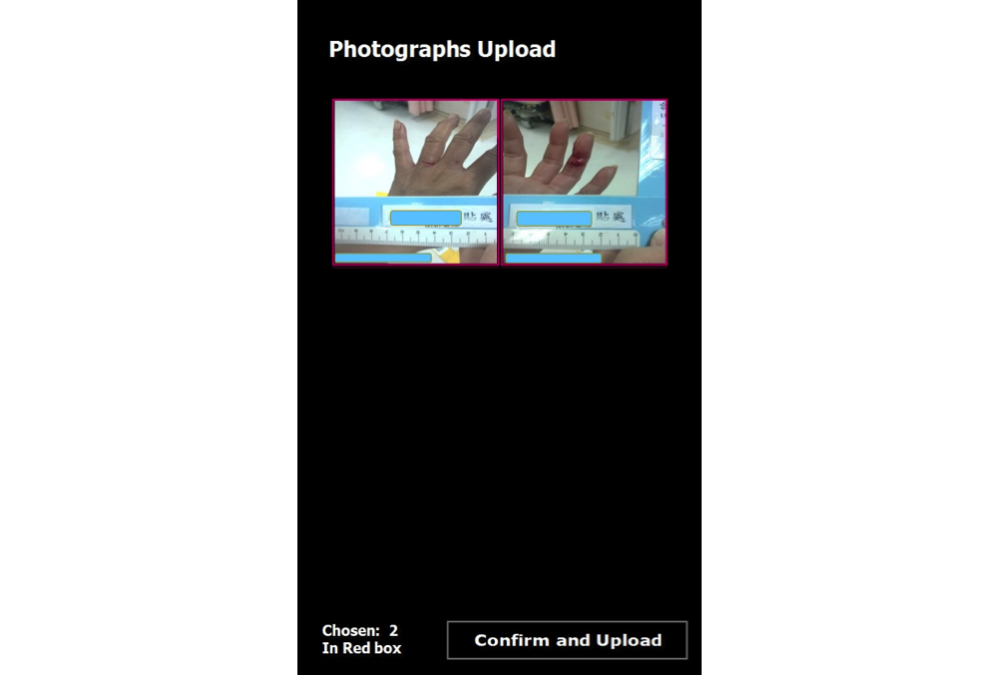
Photo selection and upload.

By using the programming language Cordova, we designed and created a purpose-built app for the Chia-Yi Christian hospital (CYCH), referred to as the *CYCH* *Fastshot*. The app is currently specific to the Android operating system and is only used for ED photography. The app has multiple functions (Figure 1), including: (1) scanning a barcode on the patient’s wrist band; (2) connecting wirelessly to the hospital information system (HIS) through an intranet network to verify patient identity (Figure 2); (3) capturing patient photos by using the phone’s built-in camera; (4) enabling convenient photograph selection (Figure 3); (5) sending the selected photos, wrapped in a data packet, to a folder on the patient’s EMR through the HIS; (6) notifying users about failed transmission through notices on the screen (the photos are stored temporarily in the app, and users can choose to either resend or restart the app to resume the upload); and (7) deleting of all the patient’s photos after securely uploading the image data packet to the EMR, due to a signal from the HIS [7].

Here, smartphones with the app were all hospital-owned and delivered encrypted photographic data packets over a Wi-Fi intranet network without using a subscriber identity module (SIM) card [13]. This absence of internet service prevented patient information from being leaked or hacked, thus maximizing patient privacy. All hospital-owned devices were tagged with the hospital’s logo and barcode to facilitate the ability to discern them from private devices and thus improve instrument management.

### Study Design

A comparative study was conducted at a busy, academic, urban ED that has approximately 100,000 visits annually. The upload efficiency of ED patient photography using digital cameras and the app was compared. In the ED, nurses serve as photographers when an appointed physician makes a request. On these occasions, another nurse on the team manages positioning and treatment of the patients with multiple or complicated wounds, ensuring that one nurse can focus entirely on only recording photos.

Here, the recruited nurses had all received standard training in ED clinical photography so that they could quickly record and identify clear photos. They also knew how to place a measurement device for scale, <1 inch from the point of the target lesion, and how to record photos with properly identifiable body parts. All the recruited nurses had more than 3 months of experience using both the camera and the app for photography.

### Data Collection

Each nurse was assigned two types of photography both before and after the app launch. An observer recorded and calculated the time spent from starting the devices to completing an upload to the EMR. The observers were a registered nurse and a research assistant who were familiar with ED nursing, the requirements of clinical photography, and the execution of the research plan. The photography processes of each group were segmented and calculated for comparison according to the time spent on each step. The entire upload operating procedure for each device is presented in Table 1.

Every computer in the nursing station was equipped with a card reader and a connector cable for the camera. The Wi-Fi intranet system was set up for data transmission from laptops, secondary monitors, and ultrasound machines in the ED. The specific devices used in this experiment were a Panasonic DMC-FH4 (digital camera) and an HTC U11 (smartphone).

**Table 1:** Photography processes for the digital camera and smartphone app groups.

Process	Digital camera group	Smartphone app group
(0) Start patient verification	N/A^a^	S0: Start app to scan the barcode on wristband
(1) Record photos	D1: Start digital camera to record photos	S1: Record photos
(2) Upload photos	D2: Move to computer and connect the DC or storage card, select photos to upload to EMR^b^	S2: Select and upload photos to EMR
(3) Delete photos	D3: Manually delete photos from storage card	N/A

^a^N/A: not applicable.

^b^EMR: electronic medical record.

### Data Analysis

Recording and uploading multiple photos of a single patient required the nurses to spend extra time on them. Thus, the cases were divided into subgroups for comparison according to the number of photos uploaded. Few cases required an upload of more than three photos, and these cases were excluded from the study as outliers. Similarly, interruptions unrelated to photography were excluded. However, delays caused by equipment failure, connection or data transmission were retained. The observers were expected to keep records and check that photos were successfully transmitted to the correct patient EMR archives. For analysis of the timing data, a two-tailed paired *t* test was performed to compare the mean values between the digital camera and smartphone app groups. The chi-square test was used to compare the distribution differences of different numbers of photos uploaded between the two groups.

## Results

Initially, 50 qualified nurses were recruited, but one resigned and another took parental leave before app implementation. Finally, 48 nurses completed camera and app photography both before and after the smartphone app implementation. Each nurse successfully recorded photographs of different patients by using digital cameras and the smartphone app. The total process time of the camera and app group was 96.3 s (SD 19.3; *P*<.001) and 24.6 s (SD 4.7; *P*<.001), respectively. The time spent on individual process segments was calculated for analysis. The process codes are listed in Table 1.

S0 (5.8 s; SD 0.9) represents the time spent in barcode scanning and identification. D1 (14.5 s; SD 5.8; *P*<.001) and S1 (11.9 s; SD 3.5; *P*<.001) represent the time spent taking photos. D2 (71.5 s; SD 17.8; *P*<.001) and S2 (6.9 s; SD 1.2; *P*<.001) represent the time to connect and select photos and uploading photos. D3 (10.3 s; SD 2.1) represents the process of manually deleting photos from the digital camera storage card. All findings are presented in Table 2.

The photography time considerably differed between the two groups. The number of photos recorded for each group is listed in Table 3. Some cases required only a single photograph; however, the majority of cases fell into the two-photo subgroup as nurses often had to record a second photo if the target body part was not sufficiently distinguishable in the first photo. There was no significant statistical difference between the digital camera and smartphone app groups in terms of the distribution of the different number of photos uploaded (X^2^_2_=.384; *P*=.83).

The data were divided into subgroups according to the number of photos recorded in both groups to enable specific process comparison. The total process time for both groups, according to the number of photos uploaded, is illustrated in Table 4.

**Table 2:** Time spent on individual segmented processes (in seconds).

Process		Minimum	Maximum	Mean (SD)	*t* test (df)	*P* value
**(0) Start patient verification**						
	Digital camera	N/A^a^	N/A	N/A	N/A	N/A
	Smartphone (S0)	4	8	5.8 (0.9)		
(**1) Record photos**					4.3 (47)	<.001
	Digital camera (D1)	7	20	14.5 (5.8)		
	Smartphone (S1)	5	19	11.9 (3.5)		
**(2) Upload photos**					25.2 (47)	<.001
	Digital camera (D2)	34	120	71.5 (17.8)		
	Smartphone (S2)	6	11	6.9 (1.2)		
**(3) Delete photos**						
	Digital camera (D3)	7	12	10.3 (1.4)		
	Smartphone	N/A	N/A	N/A		
**Total**					26.5 (47)	<.001
	Digital camera	54	152	96.3 (19.3)		
	Smartphone	15	34	24.6 (4.7)		

^a^N/A: not applicable.

**Table 3:** Subgroup categories separated by number of photos uploaded.

Number of photos uploaded	Digital camera, n	Smartphone app, n
1	14	12
2	26	29
3	8	7

**Table 4:** Average time spent in each group according to the number of photos uploaded. All values represented are in seconds.

Variable	1 photo		2 photos		3 photos	
	Digital camera	Smartphone app	Digital camera	Smartphone app	Digital camera	Smartphone app
Recording time	10	7.3	15.3	12.4	17.9	16.9
Uploading time	56.8	6.6	74.9	7	80.4	7.3
Total	77.3	19.8	100.4	25.1	108.7	30

The photography processes were isolated for comparison of the digital camera and app groups, as shown in Table 4. Although the difference in photography time for the camera and app group was small, it was significant (*P*<.001), indicating that the selection and upload led to the largest difference between the two groups (Table 4).

In 12.5% of cases (6/48), the patient photos were left undeleted on the storage card by the previous users in the camera group. Because the observer randomly initiated the experiment the camera group did not have a chance to check the digital camera in advance, which is similar to the actual conditions they’d be operating under. These six cases (88.7 s; SD 15.6) actually took a longer time than the others did (72.1 s; SD 14.3; *P*<.001) to select and upload the photos.

## Discussion

### Principal Findings

The smartphone app group (24.6 s; SD 4.7) spent almost three times less time on the entire photography process than the digital camera group (96.3 s; SD 19.3). The average time of the barcode scanning and verification process was 5.8 s (SD 0.9). The speed of wireless data transmission and verification from the HIS was fast and steady when the app device was sufficiently close to scan the barcode and nurses were proficient with its use. The barcode system has been used widely and has been effectively implemented for patient identification, medication, and blood tubing [14,15].

The time spent on isolated photography processes differed significantly between the D1 (14.5 s; SD 5.8; *P*<.001) and S1 groups (11.9 s; SD 3.5; *P*<.001). This difference was due to two factors: (1) the app had a faster autofocus function than the camera; and (2) the device startup time for the camera was included in the data.

The major difference between the two groups was in the time taken for photo selection and uploading, with D2 taking 71.5 s (SD 17.8; *P*<.001) versus S2 taking 6.9 s (SD 1.2; *P*<.001). The digital camera group took considerably more time than the smartphone app group because they had to work on the computer. Connecting the device, selecting the patient’s photos on the screen, and then uploading them to the target EMR file constituted a time-consuming process. On average, the app group spent one-tenth of the time of the camera group in this process segment.

Several types of hospital equipment transmit data over the wireless network and into the HIS, where the EMR archives are located [16]. In this study, the photos of both groups were all uploaded to the EMR archives without errors or leaks. Although the standard procedure was to manually delete the photos recorded by the digital cameras after upload, images were found on the device in 12.5% of the cases. The presence of images on the cameras may be due to negligence or time constraints for the previous users, which indicates that staff training programs in the future should include, and insist on, photo deletion. This should at least be the case in hospitals where the smartphone app method is not adopted. The photos left undeleted may not have even been uploaded to the EMR. In cases where photos remained on the camera, the user took longer in the reading and selection process (88.7 s; SD 15.6) than the average (72.1 s; SD 14.3 s; *P*<.001). The additional steps in the digital camera process and the multitasking nature of the nurses’ occupation are the likely cause of this oversight [17].

Another factor to consider is that digital camera storage cards and card readers are often damaged due to the frequent connecting and disconnecting procedures. Because smartphones transmit data wirelessly, device fatigue is not usually a factor requiring consideration. In a few cases, broken screens due to accidental dropping of the smartphone during use were observed, however, the cost-benefit ratio of the equipment and widespread availability of the devices means this damage would likely be offset easily. Ultimately, a specific study on the cost-benefit ratio of smartphone use is required.

The main benefit of using a smartphone rather than a digital camera is efficiency, particularly in a busy ED. Since all the nurses in this study now prefer the smartphone app, digital camera photography serves as an alternative, backup solution after the launch of the app in the ED.

All patient photos are strictly confidential; however, photos of patients who were victims of sexual assault are stored on a separate storage card kept in a sealed box, accessible only to the police. These special conditions were excluded from the study.

In general, the smartphone app group demonstrated superior results compared with the digital camera group because all the photos captured were processed more quickly and deleted automatically. The manual deletion process in the camera group added an extra 10.3 s to the processing time.

### Limitations

In this study, neither misfiled nor missing photos were observed in both groups. This absence of errors may be attributed to the observer effect or the small sample size. However, data from the hospital’s nurse reporting system indicated that 9-13 cases of ED missing photos or archive errors occurred annually before the app was launched. Archive errors endanger patient safety and their correction requires a large amount of time. These problems increase stress levels, frustration, and discontentment because of the high risk of medical errors [18]. As mentioned, nurses endure high levels of discontinuity in the execution of their work and frequently have to manage interruptions; these factors increase the potential for errors. Strategies that reduce the number of interruptions or devices that mitigate dangerous interruptions are required [19]. The strongest advocates for foolproof devices and systems are the nurses themselves, and they were a key driving force in development of this app [20]. The nurses’ heavy workload, with many interruptions and distractions throughout the day, also often leads to job dissatisfaction and burnout [21]. After taking photos in the ED, the camera group took several seconds to move to a nearby computer and upload the photos. However, in this short period, many nurses were interrupted or distracted by requests from patients, families, or colleagues. These incidents may explain the reason that some photos were left on the storage cards of the cameras and neither uploaded nor deleted.

The Wi-Fi connection at the hospital was strong and no transmission errors or delays were noted in the study. A strong and stable Wi-Fi network and support from the IT department was essential for the project’s success.

### Conclusion

Smartphones are becoming increasingly ubiquitous. A relatively simple photography app can add safety, security, and timeliness to a hospital’s ED. Verifying patient identity, prevention of archival errors, and ease of use are all essential aspects of a smartphone app. The improvement of patient privacy and the prevention of leaks and hacks also improve the outcome of this study. This app could potentially increase the efficiency of clinical photography, reduce the workload of nurses, and mitigate stresses caused by frequent interruptions.

## Abbreviations

CYCH: Chia-Yi Christian Hospital
ED: emergency department
EMR: electronic medical record
HCP: health care provider
HIS: hospital information system
IT: information technology
NHI: National Health Insurance
SIM: subscriber identity module
